# 制备气相色谱在挥发性成分分离中的应用研究进展

**DOI:** 10.3724/SP.J.1123.2022.04013

**Published:** 2023-01-08

**Authors:** Abulitifu MAYIRA, Zihao ZHONG, Xi BAI

**Affiliations:** 新疆师范大学化学化工学院, 新疆 乌鲁木齐 830054; College of Chemistry and Chemical Engineer, Xinjiang Normal University, Urumqi 830054, China

**Keywords:** 制备气相色谱, 挥发性化学成分, 分离, 综述, preparative gas chromatography (Prep GC), volatile compounds, separation, review

## Abstract

分析型气相色谱仪对低沸点易挥发的有机化合物展现出优异的分离性能,通过在其色谱柱末端加装馏分收集装置,建立了现代制备气相色谱(Prep GC)技术,该技术可用于挥发性成分的快速分离富集。制备气相色谱仪是由分析型气相色谱仪改装而来,其进样系统、分离系统、检测系统、馏分收集系统也在不断地优化升级,以提高目标化合物的回收效率和纯度。Prep GC与现代波谱技术(如紫外可见吸收光谱、红外吸收光谱、拉曼光谱、质谱、X射线衍射、核磁共振波谱)耦合,可对分离富集得到的目标化合物的结构进行精准确证。近年来,与Prep GC在各种挥发性成分分离中的应用相关的报道逐渐增多,展现出良好的应用前景。然而,Prep GC在分离过程中也存在着无法制备热敏性化合物、分离成本高、易引入外源性污染等问题。该文根据近年来国内外研究学者的相关研究工作,对制备气相色谱仪的结构及其在精油单体化合物、昆虫信息素、食品和植物挥发性成分、地质生物标志物及持久性环境污染物的分离等领域的应用研究进展进行综述。最后,还对Prep GC在挥发性成分分离中的应用进行了总结与展望,旨在为拓展Prep GC应用领域提供参考。

## Abstract (en)

Gas chromatography (GC) yields superior separations of low boiling point volatile compounds. Therefore, preparative gas chromatography (Prep GC) was established by combining analytical GC with a sample collection system at the end of the column, enabling the efficient isolation of the volatile components from complex matrices and their subsequent collection after GC. As Prep GC is based on an analytical gas chromatograph, its injection, separation, detection, and fraction collection systems are continuously optimized and upgraded to improve the recoveries and purities of the target compounds. Prep GC, in combination with modern spectroscopic techniques (such as UV-Vis absorption spectroscopy, infrared absorption spectroscopy, Raman spectroscopy, mass spectrometry, X-ray diffraction, and nuclear magnetic resonance spectroscopy), enables accurate structural elucidation of a target compound. Reports of the separations of various volatile components from complex matrices using Prep GC have recently increased annually, revealing promising application prospects. However, Prep GC also displays several disadvantages, such as the failure to separate thermolabile compounds, high separation costs, and the likely introduction of exogenous contamination. Based on the recent related research, this review summarizes the evolution of the structure of Prep GC and its application in isolating essential oil monomers, insect pheromones, volatile food and plant components, geological biomarkers, and persistent environmental pollutants. Finally, this review also summarizes and prospects the use of Prep GC in separating volatile components to provide a reference for the expansion of its applications.

制备色谱是一种以分离得到足量的高纯度单体化合物为目的的色谱分离技术,现已广泛用于结构解析、生物活性筛选、感官评价及分析对照品的批量制备等领域。对于无法用重结晶及蒸馏纯化的热敏性化合物,高效液相色谱是理想的分离工具。制备液相色谱可通过大直径柱结合馏分收集器在室温下分离富集热敏性单体化合物,制备规模可从mg级至kg级^[1,2]^。同时期发展起来的气相色谱技术对微量或痕量挥发性成分具有高效的分离性能,但因其分离后的馏分为气态,不易收集,制备气相色谱仪一直未能实现完全商品化。目前,文献报道的制备气相色谱仪多是在分析型气相色谱仪色谱柱的末端加装馏分收集装置改造而来,采取重复进样或大体积进样来实现μg/mg级别的目标化合物的制备^[3]^。早期制备气相色谱的馏分收集装置多为自制冷阱,收集效率较低;而商品化自动馏分收集器的问世,极大地改善了分离的效率和精准度,进一步提高了制备气相色谱仪的自动化水平。气相色谱-质谱联用技术在挥发性有机化合物的定性方面有着无法比拟的优势,其主要依靠有机化合物的保留值和标准谱库比对定性,而部分同分异构体或新化合物可能具有相同的保留值,且它们的质谱裂解规律相似,过度依赖上述两种方法容易得出错误的定性结果。利用制备气相色谱(Prep GC)结合现代波谱学技术(紫外可见吸收光谱、红外光谱、拉曼光谱、X射线衍射、质谱、核磁共振波谱等)对气相色谱-质谱联用分析中的未知物进行分离富集和结构确证则可弥补上述缺陷。随着样品的复杂程度不断增加,二维或多维Prep GC已逐渐应用于挥发性成分的分离。近年来,Prep GC已在多个领域得到广泛应用。Zuo等^[4]^在2013年通过查阅1950~2010年间发表的英文文献,对Prep GC的结构及其在精油化学成分、同位素、同分异构体、手性化合物的分离应用进行了综述;2015年,Sciarrone等^[5]^综述了2010~2014年间Prep GC的发展及应用情况,并指出Prep GC在分离纯化单体化合物的过程中,溶剂用量少,是一种绿色高效的分离技术;2017年,张海龙等^[6]^对Prep GC技术在生物标志物单体放射性同位素分析中的应用进行了综述;2021年,Kim等^[7]^综述了一维和多维Prep GC的结构及它们在昆虫信息素、药物杂质、环境污染物分离方面的应用进展。本文将结合近年来国内外研究者的相关研究工作,对制备气相色谱仪结构的演变及其多个领域的应用进展进行综述,并对其发展前景进行展望。

## 1 制备气相色谱系统

典型的制备气相色谱系统主要由进样系统、色谱柱、检测器和馏分收集系统构成(见图1)。

**图1:**
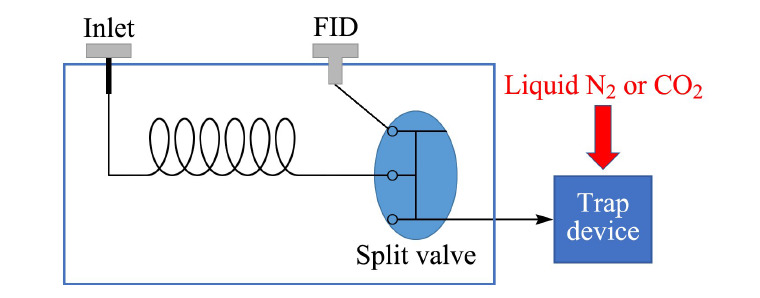
制备气相色谱示意简图^[8]^

### 1.1 进样系统

常用的制备毛细管柱气相色谱的进样方式主要有不分流进样和大体积进样,不分流进样由常规的分流/不分流进样口实现,大体积进样则通过程序升温气化进样口实现。在分析型毛细管柱气相色谱中,不分流进样主要适用于向进样口引入浓度较小的样品,以达到检测器的灵敏度要求;而对于制备型毛细管柱气相色谱,则需采取过载进样才能够提高制备效率,因此需要给样品提供足够大的气化空间。如果样品蒸气的膨胀体积远大于进样口衬管体积,则样品蒸气可能会通过反吹作用从分流出口、隔垫吹扫出口及载气管线逸散损失,由此也会造成进样口衬管的污染和分流出口捕集阱、管线的污染。衬管被污染后会造成色谱峰保留时间重现性变差,直接影响馏分收集器对色谱峰切割收集的准确度;而样品浓度过大,则会使样品溶液黏性过大,导致自动进样针发生粘连,进样序列中断。

上述问题可通过大体积进样加以克服,即可将高浓度的样品用溶剂稀释以降低黏性,同时提高进样量。当样品组分与溶剂之间的沸点之差高于100 ℃时,可采用分流模式下的程序升温进样口引入样品,此时的进样体积可达100 μL以上;同时,进样口中衬管中应有惰性石英棉或吸附剂,以便冷凝的样品组分停留,而自动进样针的推杆速率也应与溶剂在衬管中的气化速率相匹配。在将溶剂用载气从分流出口中排出后,程序升温进样口的温度按既定的升温程序急速上升,将不同沸点的样品依次引入色谱柱中进行分离,此时分流出口处于关闭状态,以防样品损失^[9]^。同时,程序升温进样口还可以作为制备液相色谱与Prep GC联用时的样品接口实现多维色谱分离^[10]^。

### 1.2 色谱柱

色谱柱在Prep GC分离中起着至关重要的作用。在制备过程中,需进行过量进样,对柱容量的要求也随之提高。填充柱作为经典气相色谱的分离装置,柱容量较大。Zuo等^[11]^以石油醚和乙酸乙酯为洗脱剂,利用硅胶柱色谱对石菖蒲精油进行预分离,收集石油醚/乙酸乙酯为20∶1时的馏分,将该馏分引入制备气相色谱,用固定相为10% OV-101的填充柱分离,进样量为5 μL,连续进样90次后,得到顺式细辛醚178 mg和反式细辛醚82 mg。王海坤^[12]^利用配有10% OV-101填充柱的制备气相色谱,分别对莪术和香附精油的化学成分进行分离;从莪术精油中分离得到5种纯度较高的单体化合物,分别是*β*-榄香烯、莪术烯、呋喃二烯酮、莪术烯醇、莪术烯酮;而从香附精油中则富集得到4种单体化合物,分别为香附子烯、*β*-芹子烯、*β*-香附酮、*α*-香附酮。谢雯燕^[13]^利用固定相为SE-30的填充柱对烟草中的4种主要生物碱进行分离富集,得到产物分别为烟碱、降烟碱、新烟草碱和假木贼碱,纯度均在80%以上。尽管填充柱的柱容量大,但其柱效较低,对复杂样品的分离度较差。因此,需要利用硅胶柱色谱作为“准一维”色谱对复杂样品进行分段,再利用配有填充柱的制备气相色谱进行纯化。

目前,涂壁毛细管柱已成为应用于分离样品的主流气相色谱柱,其柱容量较小,柱效高。基于上述优势,涂壁毛细管柱已取代填充柱成为制备气相色谱分离系统的标准配置。根据近年来的文献报道,应用于Prep GC的毛细管柱一般为低相比(*β*)的大内径毛细管柱,其规格为内径0.32 mm或0.53 mm,固定液的涂渍厚度在0.5~5 μm之间。上述规格的毛细管柱具有较高的柱容量,但其分离时间可能会相应增加。因此,可针对待分离样品的性质选用涂渍有相同极性固定液的大内径毛细管色谱柱,以提高分离制备效率。近年来,不同学者分别利用涂渍有聚乙二醇、5%二苯基-95%甲基聚硅氧烷固定液的大内径毛细管柱成功实现了对挥发性成分的分离^[14⇓-16]^。由于存在色谱峰共流出的情况,配置了大内径毛细管柱的一维Prep GC的馏出物纯度仍不理想。中心切割的多维气相色谱为复杂样品中目标化合物的分离提供了高效的解决途径,因为多维色谱的峰容量等于第一维毛细管柱的峰容量与第二维(或余下多维)毛细管色谱柱的峰容量的乘积。配置有多根大内径的多维Prep GC兼具高的柱容量和分辨率,可显著提升分离馏出物的纯度^[17⇓-19]^。涂渍高厚度固定液的大内径毛细管色谱柱在较高的分离温度下使用时,固定液会发生流失并随载气气流进入馏分收集装置,污染分离纯化的产物,致使利用红外吸收光谱和核磁共振波谱鉴定馏出产物的结构时出现干扰峰信号,不利于后续化合物结构解析鉴定^[20]^。

### 1.3 检测器

热导池检测器(thermal conductivity cell detector, TCD)和氢火焰离子化检测器(hydrogen flame ionization detector, FID)是气相色谱常用的两种检测器,TCD为非破坏型的通用型检测器,其入口可与色谱柱直接相连,出口可直通馏分收集装置,无需十字分流装置。陈占营等^[21]^建立了一套配置TCD的制备型气相色谱系统,成功地从氙氡混合样品中分离得到纯度大于85%的氙气,实现了氙样品中氡的高效去除及氙的高效制备。TCD在永久性气体分离中有独特的优势,但TCD的灵敏度较低,对痕量组分可能无响应。FID灵敏度高,但为破坏型检测器,需要在色谱柱的末端安装分流装置,引导少量组分进入检测器,而大部分样品则经传输线进入馏分收集器。王海坤^[12]^在填充柱末端加装分流器,通过控制分流器两端的限流阀,设定收集流量和FID流量之比为95∶5,成功地从莪术精油中分离到单体化合物。而对于毛细管柱来说,通行的做法是在毛细管末端加装十字分流阀,在阀的两端加装不同内径的毛细管,一端连接内径为0.1 mm的脱活毛细管进入FID,另一端连接内径为0.32 mm的惰性毛细管作为传输线进入馏分收集器。

近年来,随着联用技术的不断发展,Prep GC与质谱、核磁共振波谱仪联用也已逐渐成为研究热点。荷兰的Jeroen Kool教授课题组已成功将Prep GC与质谱、核磁共振波谱仪联用,实现了对环境污染物(如抗雄激素类物质、氯化石蜡)的分离制备和结构鉴定^[22⇓-24]^。

### 1.4 馏分收集装置

馏分收集装置位于制备气相色谱检测器的末端,其主要作用是将分离得到的高纯度气态单体化合物进行浓缩富集。目前,Prep GC的馏分收集装置主要有商品化的馏分收集器、大内径的毛细管短柱、吸附剂等。

#### 1.4.1 商品化的馏分收集器

目前,配置商品化馏分收集器的制备气相色谱仪已广泛应用于复杂样品中挥发性目标组分的快速分离^[25]^。商品化的馏分收集器的生产商全部为国外公司,各产品之间性能指标差异主要在于捕集阱的数量及冷阱温度,表1对部分商品化馏分收集器的性能指标进行了对比。

**表1:** 部分商品化馏分收集器的性能指标^[26⇓⇓⇓⇓-31]^

Manufacturer	Model	Transfer line temperature/℃	Trapping oven temperature/℃	Collection tubes			Trap cooling	
				Number	Capacity		Mode	Temperature/ ℃
Switzerland Brechbühler	Prep 9100	up to 325	-	10	1-4 mg		-	-
Germany JAS	JAS EzPrep	-	20-320	9^*^	2 mL		-	-
Japan GL Sciences	GL Sciences VPS2800	60-380	60-380	7^*^	-		stirling cooling technology	0 to -30 (forced air); 0 to -60 (cooling block surface)
Germany Gerstel	Gerstel PFC	up to 350	up to 250	7*	1 μL or 100 μL		LN_2_ or cryostatic cooling system	down to -150 (LN_2_ cooling system); down to -20 (cryostatic cooling)
France Chopper	View Prep Station	40-300	40-300	7^*^	-		peltier element cooling system	0 to ambient temperature

PFC: preparative fraction collector; LN_2_: liquid nitrogen; * including 1 waste collection tube.

其中,德国Gerstel Preparative Fraction Collector(PFC)是目前应用比较广泛的商品化制备气相色谱专用收集器,配有6个样品收集器和一个废弃物收集器。捕集阱的体积为1 μL或100 μL。为了获得最佳的化合物回收率,PFC可以适配液氮捕集阱冷却系统或循环冷浴捕集阱冷却系统^[32]^。该馏分收集器可以收集单个化合物、一系列化合物或特定类别的化合物。经过气相色谱柱高效分离的目标有机化合物单体通过十字分流阀,约有1%经内径为0.1 mm的脱活毛细管进入FID,其余99%通过内径0.32 mm的毛细管传输线进入八通阀回收装置,经微处理器控制,八通阀的切换时间可以在0.01 min内,能够可靠地收集色谱柱上间隔紧密的单个化合物。PFC可通过分析型气相色谱数百次重复进样捕获mg级别的组分,为后续利用核磁共振或红外吸收光谱等手段进行结构确证奠定基础。尽管PFC已实现气相色谱分离组分的自动收集,但对不同类型的化合物其分离条件仍需优化。Zhang等^[33]^以直链烷烃、直链脂肪酸酯、甾烷和多环芳烃等11种物理化学性质不同的标准品在制备气相色谱上的回收效率为考察指标,对Prep GC的分离富集条件进行优化。结果表明,制备气相色谱仪的最优分离条件如下:程序升温进样口的起始温度为20 ℃(低沸点样品)或80 ℃,以720 ℃/min的速率升温到350 ℃,注射速率为300 μL/min,放空流量为20 mL/min,放空时间为5 s,放空压力为68.95 kPa (10 psi),载气(氦气)流量为5 mL/min, PFC传输线和阀箱的温度分别为280 ℃。而对于捕集阱冷却系统的温度设定,则无统一标准,高沸点化合物的捕集温度为45 ℃,低沸点化合物的捕集温度为-5 ℃。

#### 1.4.2 大内径毛细管短柱

基于大内径毛细管短柱在顶空富集挥发性成分时的优异表现,其与低温冷阱耦合已成为制备气相色谱经济、高效的馏分收集装置。大内径毛细管耦合低温冷阱装置利用微板流路控制元件Dean Switch实现检测器与馏分收集流路之间的流量分配,这与Gerstel PFC利用无死体积十字分流阀分配检测器与捕集阱传输线之间的流量方式有明显的区别^[34]^。Eyres等^[35,36]^和Rühle等^[37,38]^用安捷伦微板流路控制系统Dean Switch引导目标化合物分别进入FID和大内径毛细管短柱馏分收集装置。为收到良好的富集效果,该收集装置适配有干冰捕集阱冷却系统,经气相色谱端多次重复进样后,将大内径毛细管短柱从冷阱中取出,用相似极性的溶剂提取后进行核磁共振或X-射线衍射分析。Nojima等^[39]^将40 cm长,内径为0.53 mm的毛细管柱与气相色谱分离色谱柱相连作为捕集阱,收集目标化合物。为了提高强挥发性成分的收率,外加热绝缘贮存槽用于制冷剂的投放;经过一段时间捕集,将大内径毛细管短柱中的馏分用氘代试剂洗脱入核磁样品管内进行核磁共振波谱分析。Nojima等^[40]^利用制备气相色谱分离富集C_4_~C_20_的正构烷烃、直链脂肪酸酯、直链醇标准品,以回收率为指标,考察了脱活毛细管柱、涂渍有甲基聚硅氧烷和聚乙烯醇固定液的毛细管柱的回收效率。结果显示,涂渍有甲基聚硅氧烷的毛细管柱对上述标准品的回收率为80%~100%。

#### 1.4.3 吸附剂

固体吸附剂对于样品中的痕量挥发性成分具有良好的富集作用,已被用作制备气相色谱馏分收集。Sciarrone等^[17]^将填充有2,6-二苯基对苯醚多孔聚合物(Tenax)的玻璃管置于制备气相色谱分离毛细管柱的末端作为馏分收集装置,以干冰为制冷剂,考察了正构烷烃、单萜化合物、内酯化合物在该类型制备气相色谱上的回收率。结果发现,对于低沸点的正构烷烃和单萜化合物,回收率均在95%以上;而对于高沸点的化合物,在收集玻璃管内不填充吸附剂,且不用干冰制冷的条件下,仍可获得高回收率。Ochiai等^[41]^将搅拌棒热脱附-Prep GC与嗅闻/质谱系统耦合,以Tenax玻璃管作为单通道PFC的捕集阱,增配PFC气动阀吹扫富集组分快速进入吸附管,实现对分离得到的痕量风味成分进行结构和味觉指认。采用上述装置对10 ng的15种白葡萄酒的拟香化合物(含醇类、醛类、内酯类和酚类化合物)进行模拟制备回收,回收率在85%~98%之间。Clery等^[14]^将5 mg乙基苯乙烯、二乙烯基苯共聚物(Porapak Q)作为吸附剂置于0.5 mm玻璃管作为Prep GC的馏分收集装置,其末端用截止阀与真空泵相连。利用该装置对香附精油中的主要呈香成分进行分离富集,成功得到4种含氮二萜类化合物。

## 2 制备气相色谱法的应用

### 2.1 在精油单体化合物分离中的应用

植物精油具有良好的生物活性,可用于驱蚊剂、驱虫剂及抑菌剂等,但因其沸点较低,且极易发生氧化,不能直接使用,因此需对其活性成分进行分离。早期的制备气相色谱大多配置了填充色谱柱,柱容量大,手动进样即可从精油中分离出mg级的单体化合物^[42]^;近年来有关Prep GC分离精油单体化学成分的报道多为毛细管柱Prep GC,则需配制自动进样器,以实现对精油中单体化合物的高通量制备。对于成分较为复杂的精油样品,即使使用理论塔板数较高的毛细管柱也不可能一步到位进行分离制备。因此,Prep GC分离精油组分的策略主要分为两类:一类是运用硅胶柱色谱、逆流色谱或分馏等方法对精油进行粗分,收集馏分经减压浓缩后,引入Prep GC后获得单体化合物;另一类则是运用中心切割多维气相色谱,对感兴趣的馏分进行多维纯化后获得单体化合物。Tissandié等^[43]^以愈创木油为研究对象,利用硅胶色谱柱色谱法,以石油醚/乙醚为溶剂体系进行梯度洗脱,初步得到愈创木油不同极性部位;再用硝酸银硅胶柱色谱对弱极性馏分进行二次富集,所得馏分引入制备气相色谱仪分离,最终分离得到15个新倍半萜类化合物。Niebler等^[44]^用Kugelrohr蒸馏对乳香精油进行预分离,收集沸程在125~170 ℃的馏分,经气相色谱-质谱联用法鉴定为含氧倍半萜类化合物。将该馏分经逆流色谱预分离后上制备气相色谱分离纯化,最终分离得到2个含氧倍半萜化合物,分别为莫斯德酮和莎草奥酮。Kambiré等^[45]^用硅胶柱色谱对产自科特迪瓦的翼齿六棱菊精油进行预分离,再将收集到的馏分上制备气相色谱分离得到2个新的桉叶烷型萜类化合物。Clery等^[14]^对用酸提碱沉法得到干膜莎草精油的中性部分和碱性部分,并用Flash柱色谱对上述两部分进行细分,其中中性部分经梯度洗脱所得馏分经气相色谱嗅闻系统鉴定含有目标香气成分。该部分洗脱物再经制备气相分离纯化,得到4个新的含氧倍半萜类化合物。近年来,随着多维气相色谱分析技术的发展,多维Prep GC也应运而生。此项技术的诞生,可省去庞杂的精油预分离过程,大大简化了分离纯化流程。同时,Prep GC与高效液相色谱串联后也可达到事半功倍的效果。Sciarrone等^[46]^用中心切割二维Prep GC对一种产自巴西的马鞭草破布木精油进行分离纯化,得到反式-檀香醛和反式-佛手柑油烯醛两种化合物,纯度均在90%以上。Pantò等^[10]^分别采用中心切割三维制备气相色谱法和液相色谱-中心切割二维制备气相色谱联用法对檀香木精油进行分离纯化,得到顺式-*α*-檀香醇等化合物,结果发现液相色谱-中心切割二维制备气相色谱联用法的制备效率较高,且能达到mg级制备水平。尽管Prep GC在精油单体化合物的分离中应用广泛,但受制于进样口的高温,精油中的热敏感性化合物(如异莪术呋喃二烯、驱虫蛔萜、吉玛烷型倍半萜等)无法使用Prep GC进行制备^[47⇓⇓⇓⇓-52]^。近几年来,Prep GC在精油单体化合物分离中的应用研究进展见表2。

**表2:** 制备气相色谱在精油单体化合物分离中的应用^*^

Sample	Target compounds or compound types	GC column	Trapping method	Refs.
Essential oil of Juglans regia	11-hydroxy-2,4-cycloeudesmane	HP-Innowax (30 m×0.53 mm× 1.0 μm)	Gerstel PFC	[53]
Essential oil of Crinitaria tatarica (Less.) Sojak	Z-artemidin, E-artemidin	HP-Innowax (30 m×0.53 mm× 1.0 μm)	Gerstel PFC	[54]
Guaiacwood oil	guaiane oxides	Supelcowax (30 m×0.53 mm× 0.5 μm)	Gerstel PFC	[55,56]
Peppermint oil	menthol, menthone	DB-5 (15 m×0.32 mm×1 μm)	Mega-bore capillary in a cryotrap	[57]
Peppermint oil	limonene, eucalyptol, menthone, isomenthone, neomenthol, menthol, menthyl acetate, menthofuran	InertCap PureWAX (60 m×0.32 mm×0.50 μm)	VPS2800 automated fraction collector	[58]
Essential oil of Prangos heyniae	eudesmane type sesquiterpene ketone	HP Innowax (30 m×0.53 mm× 1.0 μm)	Gerstel PFC	[59]
Essential oil of Laggera pterodonta	eudesman-4a-ol epoxides	ZB-5 (30 m×0.53 mm×3.0 μm)	Gerstel PFC	[60]
Essential oil of Ruta graveolens L.	terpenoids, lipid, alcohol, ketones compounds	DB-5 (30 m×0.53 mm×1.0 μm)	Gerstel PFC	[61]
Lavender essential oil	terpenoids, lipid compounds	DB-5 (30 m×0.53 mm×1.0 μm)	Gerstel PFC	[62]
Essential oil of Elionurus tristis Hack.	2-epi-ziza-5-en-2-ol,2-epi-prezizaan- 7-ol,7-epi-khusian-2-ol, antsorenone, 4,8-di-epi-acorone	column 1: ZB-5 (30 m×0.53 mm×3.0 μm); column 2: Supelcowax (30 m×0.53 mm×0.5 μm)	Gerstel PFC	[63]

* Injection mode: automated splitless injection.

### 2.2 在昆虫信息素分离中的应用

昆虫信息素是由昆虫分泌到体外,能在同种个体间或种间产生生理或行为反应的化学物质,对昆虫的定向、召唤、交尾、产卵、聚集、追踪、告警、防御以及种间识别等行为具有重要的作用。信息素常为昆虫体内分泌的几种结构类似的化合物的混合物,含量为ng或pg级,大多具有挥发性,毒性低,可用于制备诱捕剂或迷向剂来防治害虫。近年来,Prep GC在昆虫信息素的制备分离和结构鉴定中有较为广泛的利用。

为了防治棉粉蚧对植物的伤害,Tabata等^[64]^采用吹扫捕集法富集未交配的雌性棉粉蚧释放的性信息素,并以对雄性棉粉蚧的引诱率为活性分离导向,综合运用经典柱色谱、制备液相色谱和Prep GC等分离技术,得到高引诱率(100%)的单体化合物。经高分辨质谱和核磁共振波谱鉴定,确证该化合物为(1*R*)-(2,2-二甲基-3-环丁基)-甲基-异戊烯酸酯,该化合物具有开发为棉粉蚧诱杀剂的潜力。Tabata等^[65]^采用吹扫捕集法富集雌性新菠萝灰粉蚧分泌的性信息素,并以雄性新波罗灰粉蚧的诱捕数量为活性分离导向,综合运用硅胶柱色谱、制备液相色谱和Prep GC等分离手段,获得了一种高引诱活性的的单萜化合物,具有与薰衣草醇相似的结构。经高分辨质谱和核磁共振波谱鉴定,确证该化合物为(*E*)-2-异丙基-5-甲基-3,5-二烯基己醇乙酸酯。Tabata等^[66]^运用吹扫捕集法富集雌性菠萝粉蚧的挥发性成分,综合运用制备液相色谱、Prep GC等手段,获得了一种刺激雌性菠萝粉蚧进行孤雌生殖的单萜化合物,经高分辨质谱和核磁共振波谱鉴定,确证该化合物为(2)-(对-1,2-二甲基-3-亚甲基环戊基)乙醛。Rahmani等^[67]^利用Prep GC对雄性云杉四眼小蠹分泌的信息素进行分离,得到两个关键活性化合物分别为:(+)-(1*R*,2*S*)-诱杀烯醇和(-)-(*R*)-松油烯-4-醇。通过对两种化合物进行复配,进行林间诱捕雌性云杉四眼小蠹实验,发现(+)-(1*R*,2*S*)-诱杀烯醇在信息素的功能起效中起主导作用,而(-)-(*R*)-松油烯-4-醇则居于从属地位。Millar等^[68]^以HayeSep^®^ Q为吸附剂,运用吹扫捕集法富集雄性长刺刺腿天牛分泌的挥发性物质,经二氯甲烷洗脱后,上制备气相色谱分离,得到对长刺刺腿天牛具有专属特异性诱捕作用的化合物,经核磁共振波谱和质谱鉴定,确证该化合物为(2*E*,6*Z*,9*Z*)-2,6,9-十五碳三烯醛。Xu等^[69]^以SuperQ为吸附剂,运用吹扫捕集法富集未交配的雌性微红盘绒茧蜂分泌的信息素,经二氯甲烷洗脱后,进行Prep GC分离,得到影响微红盘绒茧蜂交配的性信息素的化合物,经质谱鉴定为庚醛。象白蚁与进入其巢穴的寄居白蚁间互不接触,且能够和平共处,二者之间应存在种间识别的信息素。Jirošová等^[70]^用正己烷提取象白蚁头部分泌物,经Prep GC分离结合电生理研究,分离出两个酯类化合物,分别为(3*Z*,6*Z*)-二十烷二烯醇脂肪酸酯、十二烷醇脂肪酸酯,经活性测试确认上述两个化合物为种间识别信息素,证实了上述假说。

### 2.3 在食品和植物挥发性成分分离中的应用

挥发性成分分离分析是香料香精、食品风味研究的先导,通过Prep GC结合嗅觉探测技术对挥发性成分进行分离和感官评价,能够发现关键风味物质,有助于提高食品味觉和口感,为食品质量控制提供依据。

香气是酒类的独特特征,关键呈香组分是酿酒工艺优化和质量控制的重要参考指标。Pons等^[71]^利用Prep GC结合嗅觉探测技术从法国波尔多红葡萄酒中分离出具有鲜李香气味的特征化合物辣薄荷酮。Siebert等^[72]^利用Prep GC结合嗅觉探测对制备液相色谱预分离的苏特恩白葡萄甜酒和维欧尼葡萄酒中的香气成分分段捕集,进行香气重组和缺失实验,结果发现,顺式-橡木内酯、丁香酚、*γ*-壬内酯和2-壬烯-4-内酯是“腐橘味”苏特恩白葡萄甜酒的关键香气贡献组分;而芳樟醇、*α*-松油醇、香叶醇和苯甲醛为“甜杏味”维欧尼葡萄酒的关键香气贡献组分,为上述两种法式葡萄酒的酿制工艺的优化提供了参考。酒类的挥发性物质是消费者的重要感官成分之一,直接影响着其市场认可度。啤酒化发酵过程中产生的含硫化合物2-巯基-3-甲基-1-丁醇,具有低香气阈值,常带有洋葱气味,破坏了啤酒的口感。Noba等^[16]^运用Prep GC从发酵后的啤酒花汁提取物中分离出了形成2-巯基-3-甲基-1-丁醇的前体物质,经核磁共振波谱和质谱鉴定为2,3-表-3-甲基丁醛,并阐明了2-巯基-3-甲基-1-丁醇在啤酒花汁发酵过程中的形成机制,提出了改进发酵工艺的路径,从而抑制硫化物的产生,改善啤酒口感。

植物中的挥发性成分可在一定程度上反映其生理活动过程,Prep GC可快速对其进行分离。Kroener等^[73]^利用Prep GC对不同品种辣根中的辛辣味成分进行分离富集,结合二维气相色谱-质谱联用/嗅闻探测技术和波谱技术对纯化后的化合物进行结构鉴定,揭示了不同品种的辣根中共有的低香气阈值的成分为(3*S*,3*aS*,7*aR*)-葡萄酒内酯和3-异丙基-2-甲氧基吡嗪。Maia等^[74]^对天南星科的3种夜间开花植物花蕾顶空挥发性成分进行吹扫捕集,经Prep GC分离,得到对授粉昆虫石雕甲虫具有引诱作用的4种挥发性化合物分别为脱氢茉莉酮、异茉莉醇、乙酸异茉莉酯和(*E*)-4,8-二甲基壬-1,3,7-三烯-5-醇乙酸酯。Andersen等^[75]^将调控萜类合成酶的基因从毒萝卜新品种*Thapsia laciniata* Rouy提取并插入烟草基因中,利用Prep GC从含有TlTPS509萜类合成酶的烟草叶中分离得到了愈创木醇和布藜醇,阐明了毒萝卜*Thapsia laciniata* Rouy中调控产生愈创木醇和布藜醇的酶为TlTPS509萜类合成酶。

### 2.4 在地质生物标志物分离中的应用

地质生物标志物是地质体中能够提供有机质生物输入源、沉积和成岩过程中的环境条件等信息的特征有机化合物,这些有机化合物虽然经历了成岩、成土等地质作用影响,但其仍继承了先驱物的基本碳骨架,具有重要的地球化学研究价值^[76]^。地质生物标志物在自然环境中的含量很低,需要通过有机溶剂进行抽提富集后,经硅胶柱色谱梯度淋洗预分离后,再利用Prep GC纯化才能获得高纯度的目标地质生物标志物,用于结构鉴定。Wang等^[77]^利用硅胶柱色谱从多米尼加琥珀萃取物中分离得到直链烃馏分,该馏分再经Prep GC富集纯化,得到反映琥珀形成时期气候、植被等地理信息的标志物,经核磁共振波谱、质谱和电子圆二色谱鉴定其绝对构型为15-降-克罗-(3,12)二烯。该化合物结构与豆科孪叶豆属植物分泌的特征化合物克罗烯酸的骨架结构相似。据此,从化合物分子结构上证实了多米尼加琥珀来自豆科树脂分泌物的论断。Zhang等^[78]^利用硅胶柱色谱结合Prep GC从茂名油页岩非烃馏分中分离富集单体化合物,经核磁共振波谱、红外光谱、质谱等鉴定,确认出两个含氧的新生物标志物,分别为5,9-二甲基-6-异丙基-2-十酮和4,9,11-三甲基-6-异丙基-2-十二酮。Liao等^[79,80]^利用硅胶柱色谱结合Prep GC,对茂名油页岩中的丛粒藻烷类化合物进行分离纯化,经核磁共振、红外光谱、质谱等仪器分析手段鉴定,确认出一个新的C_33_丛粒藻烷酮和两个新的高支链烷烃,分别为C_33_-丛粒藻烷-24-酮,2,3,6,7,10,12,15,16,19,20-十甲基-10-乙基-二十一烷(C_33_-新丛粒藻烷), 2,3,6,7,10,12,15,16,19,20-十甲基二十一烷(两个非对映异构体,C_31_-丛粒藻烷),同时阐明了上述化合物在成岩过程中的可能形成机制。

碳是地球生物圈中分布最广的元素,^14^C是碳元素中一种具有放射性的同位素,可发生*β*衰变,半衰期为5730年,是理想的示踪指标。基于此建立的总有机物的放射性碳同位素特征分析方法,能够根据^14^C的衰变规律(碳龄)揭示自然界中有机碳的迁移、转化和埋藏特征。但自然界中总有机碳的组成和来源复杂,各有机化合物的碳龄不尽相同,使得总有机物的放射性碳的同位素组成掩盖了众多的物源信息,难以进行碳源分析。生物标志物单体放射性碳同位素分析技术(compound-specific radiocarbon analysis, CSRA)可提供单一生物标志物的碳源信息,有效解决上述瓶颈问题。该方法由英国有机地球化学家Timothy I. Eglinton提出^[81]^,主要是利用Prep GC技术从环境样品中分离出目标生物标志物单体,再经离线的加速器质谱仪测定mg级的单体化合物中的^14^C含量,对自然和人类活动产生的有机碳来源进行解析研究。利用该技术,Eglinton团队^[82]^还成功分离出了新不列颠海沟水深在8225、5920、4670和4130 m等4处采集的柱状沉积物中的正构烷烃和脂肪酸,并进行^14^C定年,阐明了新不列颠海沟的碳源主要来自巴布亚新几内亚森林土壤的有机质;Ausín等^[83]^利用硅胶柱色谱、Prep GC分别从葡萄牙伊比利亚边缘海底岩芯样品中分离出烯酮和长链脂肪酸,并进行^14^C定年,揭示了末次盛冰期以来北欧海次表层温度的变化演进趋势。Gierga等^[84]^利用Prep GC技术从瑞士肖松尼湖底部沉积物中分离出正构烷烃单体,并进行^14^C定年,据此推断出史前时期肖松尼湖周边植被变化及早期人类活动对肖松尼湖周边陆上环境影响。Eglinton团队^[85]^还利用Prep GC从考古文物中分离富集得到高纯度生物标志物,并对生物标志物中的^14^C水平进行分析,为文物断代定年奠定基础;但在CSRA技术应用过程中,Prep GC的毛细管色谱柱固定液涂层流失、洗脱捕集阱馏分的有机溶剂的不完全脱除、商品化溶剂中的稳定剂等都会引入外源性的碳污染^[86]^,会给微量样品^14^C水平分析结果带来正误差,需加以扣除;为应对上述问题,Eglinton团队^[20]^将Gerstel自动馏分收集器的捕集阱改为带玻璃棉的中空石英管,无需溶剂富集馏分,避免从捕集溶剂中引入外源碳,获得良好的分析效果。

基于Prep GC的CRSA技术还可用于解析人类活动对地球环境影响的重要标志化合物(多环芳烃、二元羧酸)来源^[87]^。押淼磊等^[88,89]^建立了单体多环芳烃的二维Prep GC分离和收集方法,并从海水中成功分离得到高纯度的芴、菲+蒽、荧蒽和芘,进行^14^C定年,应用于台湾海峡海水中多环芳烃的来源解析。中国科学院广州地球化学所的张干课题组^[90,91]^结合丁酯化衍生技术,利用Prep GC技术,成功地从有机气溶胶中分离出二元羧酸单体,进行^14^C定年,并应用于中国几个典型城市的大气液相过程前体物来源分析。

### 2.5 在持久性环境污染物毒性研究中的应用

现有的Prep GC大多依靠冷阱对单体化合物进行捕集,气态分析物从高温管线中直接流入低温捕集阱中易形成气溶胶,造成损失;且分离纯化馏分个数少,无法进行后续的活性筛选分析。荷兰阿姆斯特丹自由大学Jeroen Kool教授课题组^[22,24,92]^对现有气相色谱仪流路进行改造,分离组分经两个Y形分离器分流后,一路进入FID或质谱检测器,另一路被恒流泵输入的溶剂捕集后经多功能采样器顺序送入96或384孔板,实现了对馏分的实时分离收集。96或384孔板中的馏分再进行离线生物活性筛选(如抑制乙酰胆碱酯酶活性筛选、雄性激素受体介导酶报告基因筛选),分离出高毒性的持久性环境污染物,现已将该技术成功应用于环境中农药残留和内分泌干扰物的筛查。近期,Jeroen Kool教授课题组^[23]^将上述分离平台与核磁共振波谱仪离线联用,成功实现了对复杂环境污染氯化石蜡的筛查。但上述分离平台的结构改造对仪器设备配置要求较高,还难以实现大规模的商品化应用。

## 3 总结与展望

本文对Prep GC的结构及其在挥发性成分分离中的应用进行了综述。根据Prep GC的结构,采用优化的分离参数(进样方式、色谱柱类型、检测器类型、捕集阱冷却温度),才能精准获得高纯度的目标化合物。Prep GC与现代波谱学技术(紫外可见吸收光谱、红外光谱、拉曼光谱、X射线衍射、质谱、核磁共振波谱等)耦合,可实现分离单体化合物结构的精准确证;Prep GC技术已实现对精油单体化合物、昆虫信息素、食品和植物中挥发性成分、地质生物标志物以及环境污染物等的高效分离富集,在不同领域展现出良好应用前景。

Prep GC作为挥发性成分分离的有力工具,尚存在一些不足。首先,Prep GC无法对热敏性化合物进行分离制备。部分热敏性化合物在高温进样口即发生裂解,需考虑建立特殊的气相色谱进样方法加以解决;其次,Prep GC的馏分捕集阱数量少,体积小,收集时需要耗费大量制冷剂(液氮或干冰),回收率低,需通过对各种类型化合物对照品的预分离来优化捕集参数;最后,Prep GC分离过程中所用溶剂、色谱柱固定液的涂层流失都会引入外源性干扰,对分离纯化得到的单体化合物的结构确证带来困扰,需采取措施对这部分物质加以去除。由此可见,Prep GC在挥发性成分分离应用领域中尚有许多可探究的空间。

## References

[1] Wang W, He T Y, Lan T, et al. Chinese Journal of Chromatography, 2019, 37(11): 1193 31642272 10.3724/SP.J.1123.2019.05024

[2] Li H J, Chen Q. Chinese Journal of Chromatography, 2018, 36(10): 1061 30378367 10.3724/SP.J.1123.2018.06001

[3] Wang H K, Yang F Q, Xia Z N. Chemistry, 2011, 74(1): 3

[4] Zuo H L, Yang F Q, Huang W H, et al. J Chromatogr Sci, 2013, 51(7): 704 23592825 10.1093/chromsci/bmt040

[5] Sciarrone D, Pantò S, Ragonese C, et al. TrAC-Trends Anal Chem, 2015, 71: 65

[6] Zhang H L, Tao S Q, Yu M, et al. Advances in Earth Science, 2017, 32(11): 1193

[7] Kim L, Marriott P J. Gas Chromatography. 2nd ed. Amsterdam: Elsevier, 2021: 487

[8] Kim L, Tuck K L, Marriott P J. Reference Module in Chemistry, Molecular Sciences and Chemical Engineering. Amsterdam: Elsevier, 2014: 1

[9] Hübschmann H J. Handbook of GC/MS:Fundamentals and Applications. 3rd ed. Weinheim: WILEY-VCH, 2008: 123

[10] Pantò S, Sciarrone D, Maimone M, et al. J Chromatogr A, 2015, 1417: 96 26410184 10.1016/j.chroma.2015.09.039

[11] Zuo H, Yang F, Zhang X, et al. J Anal Methods Chem, 2012: 402081 22448339 10.1155/2012/402081 PMC3303141

[12] Wang H K. [MS Dissertation]. Chongqing: Chongqing University, 2011: 27

[13] Xie W Y. [MS Dissertation]. Shanghai: East China University of Science and Technology, 2010: 20

[14] Clery R A, Cason J R, Zelenay V. J Agric Food Chem, 2016, 64(22): 4566 27219519 10.1021/acs.jafc.6b00680

[15] Garcia G, Tissandié L, Filippi J J, et al. Molecules, 2017, 22(6): 921 28574456 10.3390/molecules22060921 PMC6152735

[16] Noba S, Yako N, Sakai H, et al. Food Chem, 2018, 255: 282 29571478 10.1016/j.foodchem.2018.02.092

[17] Sciarrone D, Pantò S, Ragonese C, et al. Anal Chem, 2012, 84(16): 7092 22835067 10.1021/ac3013829

[18] Sciarrone D, Pantò S, Rotondo A, et al. Anal Chim Acta, 2013, 785: 119 23764452 10.1016/j.aca.2013.04.069

[19] Sciarrone D, Pantò S, Tranchida P Q, et al. Anal Chem, 2014, 86(9): 4295 24725161 10.1021/ac404078u

[20] Casanova E, Knowles T D J, Williams C, et al. Anal Chem, 2018, 90(18): 11025 30118604 10.1021/acs.analchem.8b02713

[21] Chen Z Y, Liu S J, Wang J L, et al. Atomic Energy Science and Technology, 2016, 50(11): 1949

[22] Jonker W, Zwart N, Stöckl J B, et al. Talanta, 2017, 168: 162 28391837 10.1016/j.talanta.2017.02.067

[23] Van Mourik L M, Janssen E, Breeuwer R, et al. Anal Chem, 2021, 93(15): 6158 33832223 10.1021/acs.analchem.1c00049 PMC8153385

[24] Pieke E, Heus F, Kamstra J H, et al. Anal Chem, 2013, 85(17): 8204 23919657 10.1021/ac401384q

[25] Sciarrone D, Pantò S, Donato P, et al. J Chromatogr A, 2016, 1475: 80 27863713 10.1016/j.chroma.2016.11.013

[26] Xu G W. Handbook of Analytical Chemistry:Part 5, Gas Chromatography Analysis. 3rd ed. Chemical Industry Press: Beijing, 2016: 22

[27] Brechbühler A G. Prep 9100-State of the Art Fraction Collector for GC. [2022-04-13]. https://www.brechbuehler.ch/PREP-9100-Series.846.0.html?msclkid=68b916a1cf7e11ecab8ca0fa199eeb0ehttps://www.brechbuehler.ch/PREP-9100-Series.846.0.html?msclkid=68b916a1cf7e11ecab8ca0fa199eeb0e

[28] Joint Analytical Systems GmbH. Preparative Fraction Collector (EzPrep). [2022-04-13]. https://www.jas.de/en/products/jas/ezprep/?msclkid=00d560d2cf8211eca1250cb1577a59edhttps://www.jas.de/en/products/jas/ezprep/?msclkid=00d560d2cf8211eca1250cb1577a59ed

[29] GL Sciences. GC Fraction Collector View Prep Station VPS2800. [2022-04-13]. https://www.glsciences.com/product/gc_devices/gc_fraction_collector/00932.html?msclkid=35278553cf8411ecb7a1cc872fbfb9cahttps://www.glsciences.com/product/gc_devices/gc_fraction_collector/00932.html?msclkid=35278553cf8411ecb7a1cc872fbfb9ca

[30] Gerstel Company. GC Preparative Fraction Collector-PFC. [2022-04-13]. https://www.gerstelus.com/products/pfc/https://www.gerstelus.com/products/pfc/

[31] CHOPPER Company. CHOPPER Extreme Precision Preparative GC Station View Prep Station. [2022-04-13]. https://www.madeforindustry.com/catalog_chopper-extreme-precision-preparative-gc-station-v_3c5bbd980e5448.htmlhttps://www.madeforindustry.com/catalog_chopper-extreme-precision-preparative-gc-station-v_3c5bbd980e5448.html

[32] Novaes F J M, Marriott P J. J Chromatogr A, 2021, 1644: 462135 33839448 10.1016/j.chroma.2021.462135

[33] Zhang X, Zhao L, Wang Y, et al. J Sep Sci, 2013, 36(13): 2136 23625706 10.1002/jssc.201300088

[34] Sharif K M, Chin S T, Kulsing C, et al. TrAC-Trends Anal Chem, 2016, 82: 35

[35] Eyres G T, Urban S, Morrison P D, et al. Anal Chem, 2008, 80(16): 6293 18646864 10.1021/ac8007847

[36] Eyres G T, Urban S, Morrison P D, et al. J Chromatogr A, 2008, 1215(1/2): 168 19027909 10.1016/j.chroma.2008.10.102

[37] Rühle C, Eyres G T, Urban S, et al. J Chromatogr A, 2009, 1216(30): 5740 19541321 10.1016/j.chroma.2009.06.006

[38] Ruhle C P, Niere J, Morrison P D, et al. Anal Chem, 2010, 82(11): 4501 20441219 10.1021/ac100417h

[39] Nojima S, Kiemle D J, Webster F X, et al. PLoS One, 2011, 6(3): e18178 21464906 10.1371/journal.pone.0018178 PMC3065492

[40] Nojima S, Apperson C S, Schal C. J Chem Ecol, 2008, 34(3): 418 18297362 10.1007/s10886-008-9437-z

[41] Ochiai N, Sasamoto K. J Chromatogr A, 2011, 1218(21): 3180 21081238 10.1016/j.chroma.2010.10.027

[42] Starkenmann C, Cayeux I, Brauchli R, et al. J Agric Food Chem, 2011, 59(2): 677 21190364 10.1021/jf103989j

[43] Tissandié L, Brevard H, Belhassen E, et al. J Chromatogr A, 2018, 1573: 125 30245071 10.1016/j.chroma.2018.08.050

[44] Niebler J, Zhuravlova K, Minceva M, et al. J Nat Prod, 2016, 79(4): 1160 27010489 10.1021/acs.jnatprod.5b00836

[45] Kambiré D A, Yapi A T, Boti J B, et al. Nat Prod Res, 2020, 34(19): 2765 30908078 10.1080/14786419.2019.1586701

[46] Sciarrone D, Giuffrida D, Rotondo A, et al. J Chromatogr A, 2017, 1524: 246 29030035 10.1016/j.chroma.2017.10.007

[47] Mahanta B P, Sut D, Kemprai P, et al. Phytochem Analysis, 2020, 31(1): 28 10.1002/pca.2863 31243828

[48] Mahanta B P, Sut D, Lal M, et al. J Essent Oil Res, 2021, 33(3): 240

[49] Mahanta B P, Bora P K, Kemprai P, et al. Food Res Int, 2021, 145: 110404 34112407 10.1016/j.foodres.2021.110404

[50] Cavalli J F, Tomi F, Bernardini A F, et al. Phytochem Analysis: An Int J Plant Chem Biochem Tech, 2004, 15(5): 275 10.1002/pca.761 15508830

[51] Maggi F, Papa F, Giuliani C, et al. Flavour Frag J, 2015, 30(2): 139

[52] Ouattara Z A, Boti J B, Ahibo A C, et al. Phytochem Analysis, 2013, 24(6): 574 10.1002/pca.2435 23592386

[53] Köroglu A, Baldemir A, Özek G, et al. Nat Prod Commun, 2016, 11(10): 1421 30549590

[54] Özek G, Ishmuratova M, Tabanca N, et al. J Sep Sci, 2012, 35(5/6): 650 22331842 10.1002/jssc.201100950

[55] Tissandie L, Gaysinski M, Brévard H, et al. J Nat Prod, 2017, 80(2): 526 28195478 10.1021/acs.jnatprod.6b01068

[56] Tissandié L, Viciana S, Brevard H, et al. Phytochemistry, 2018, 149: 64 29477626 10.1016/j.phytochem.2018.02.007

[57] Park H E, Yang S O, Hyun S H, et al. J Sep Sci, 2012, 35(3): 416 22213698 10.1002/jssc.201100670

[58] Zigon N, Kikuchi T, Ariyoshi J, et al. Chem-Asian J, 2017, 12(10): 1057 28382777 10.1002/asia.201700515

[59] Özek G, Bedir E, Tabanca N, et al. Open Chem, 2018, 16(1): 453

[60] Kambiré D A, Boti J B, Filippi J J, et al. Nat Prod Res, 2019, 33(14): 2109 29865894 10.1080/14786419.2018.1482893

[61] Li Y, Dong G, Bai X, et al. Nat Prod Res, 2021, 35(21): 4202 32336143 10.1080/14786419.2020.1756798

[62] Dong G, Bai X, Aimila A, et al. Molecules, 2020, 25(14): 3166 32664436 10.3390/molecules25143166 PMC7397202

[63] Garcia G P, Sutour S, Rabehaja D, et al. Phytochemistry, 2019, 162: 29 30851508 10.1016/j.phytochem.2019.02.012

[64] Tabata J, Ichiki R T. J Chem Ecol, 2016, 42(11): 1193 27771797 10.1007/s10886-016-0783-y

[65] Tabata J, Ichiki R T. J Chem Ecol, 2015, 41(2): 194 25618324 10.1007/s10886-015-0545-2

[66] Tabata J, Ichiki R T, Moromizato C, et al. J R Soc Interface, 2017, 14(128): 20170027 10.1098/rsif.2017.0027 PMC5378144 28250102

[67] Rahmani R, Wallin E A, Viklund L, et al. J Chem Ecol, 2019, 45(4): 356 30796678 10.1007/s10886-019-01056-6 PMC6477006

[68] Millar J G, Mitchell R F, Meier L R, et al. J Chem Ecol, 2017, 43(11): 1056 29151153 10.1007/s10886-017-0905-1

[69] Xu H, Zhou G, Dötterl S, et al. J Chem Ecol, 2019, 45(7): 559 30924035 10.1007/s10886-019-01066-4

[70] Jirošová A, Sillam-Dussès D, Kyjaková P, et al. J Chem Ecol, 2016, 42(10): 1070 27639394 10.1007/s10886-016-0756-1

[71] Pons A, Lavigne V, Darriet P, et al. Food Chem, 2016, 206: 191 27041315 10.1016/j.foodchem.2016.03.064

[72] Siebert T E, Stamatopoulos P, Francis I L, et al. J Chromatogr A, 2021, 1637: 461803 33383243 10.1016/j.chroma.2020.461803

[73] Kroener E M, Buettner A. Food Chem, 2017, 232: 455 28490098 10.1016/j.foodchem.2017.04.042

[74] Maia A C D, Grimm C, Schubert M, et al. J Chem Ecol, 2019, 45(2): 204 30229355 10.1007/s10886-018-1018-1 PMC6469606

[75] Andersen T B, Rasmussen S A, Christensen S B, et al. Phytochemistry, 2019, 157: 168 30412824 10.1016/j.phytochem.2018.10.027

[76] Sheng G Y, Lu H, Peng P A. Geochimica, 2018, 47(2): 113

[77] Wang Y, Jiang W, Feng Q, et al. Org Geochem, 2017, 113: 90

[78] Zhang X, Lu H, Liao J, et al. J Sep Sci, 2017, 40(3): 813 27925402 10.1002/jssc.201600951

[79] Liao J, Lu H, Feng Q, et al. Org Geochem, 2018, 124: 103

[80] Liao J, Zhang Y J, Lu H, et al. Geochimica, 2018, 47(2): 134

[81] Eglinton T I, Aluwihare L I, Bauer J E, et al. Anal Chem, 1996, 68(5): 904 21619188 10.1021/ac9508513

[82] Xiao W, Xu Y, Haghipour N, et al. Chem Geol, 2020, 533: 119446

[83] Ausín B, Magill C, Haghipour N, et al. Earth Planet Sc Lett, 2019, 515: 38

[84] Gierga M, Hajdas I, van Raden U J, et al. Quaternary Sci Rev, 2016, 144: 123

[85] Casanova E, Knowles T D J, Bayliss A, et al. J Archaeol Sci, 2022, 137: 105528

[86] Casanova E, Knowles T D J, Williams C, et al. Anal Chem, 2017, 89(13): 7090 28557447 10.1021/acs.analchem.7b00987

[87] Zhang G, Liu J, Li J, et al. Funda Res, 2021, 1(3): 306

[88] Ya M L. [PhD Dissertation]. Xiamen: Xiamen University, 2018: 31

[89] Ya M L, Wu Y, Xu L, et al. Water Res, 2021, 198: 117134 33901842 10.1016/j.watres.2021.117134

[90] Xu B Q. [PhD Dissertation]. Guangzhou: University of Chinese Academy of Sciences (Guangzhou Institute of Geochemistry Chinese Academy of Sciences), 2021: 27

[91] Xu B, Cheng Z, Gustafsson Ö, et al. Environ Sci Tech Let, 2021, 8(2): 135

[92] Jonker W, Clarijs B, de Witte S L, et al. J Chromatogr A, 2016, 1462: 100 27485151 10.1016/j.chroma.2016.07.068

